# Genome-Wide Identification of GRAS Gene Family and Their Responses to Abiotic Stress in *Medicago sativa*

**DOI:** 10.3390/ijms22147729

**Published:** 2021-07-20

**Authors:** Han Zhang, Xiqiang Liu, Xuemeng Wang, Ming Sun, Rui Song, Peisheng Mao, Shangang Jia

**Affiliations:** 1 College of Grassland Science and Technology, China Agricultural University, Beijing 100193, China; hanzhang003@cau.edu.cn (H.Z.); b20183040361@cau.edu.cn (X.L.); xuemeng.wang@cau.edu.cn (X.W.); sunmiir@cau.edu.cn (M.S.); ruisong2021@cau.edu.cn (R.S.); 2 Key Laboratory of Pratacultural Science, Beijing Municipality, China Agricultural University, Beijing 100193, China

**Keywords:** *Medicago sativa*, GRAS genes, phylogeny, salinity and drought stresses, expression

## Abstract

Alfalfa (*Medicago sativa*) is a high-quality legume forage crop worldwide, and alfalfa production is often threatened by abiotic environmental stresses. GRAS proteins are important transcription factors that play a vital role in plant development, as well as in response to environmental stress. In this study, the availability of alfalfa genome “Zhongmu No.1” allowed us to identify 51 GRAS family members, i.e., MsGRAS. MsGRAS proteins could be classified into nine subgroups with distinct conserved domains, and tandem and segmental duplications were observed as an expansion strategy of this gene family. In RNA-Seq analysis, 14 MsGRAS genes were not expressed in the leaf or root, 6 GRAS genes in 3 differentially expressed gene clusters were involved in the salinity stress response in the leaf. Moreover, qRT-PCR results confirmed that MsGRAS51 expression was induced under drought stress and hormone treatments (ABA, GA and IAA) but down-regulated in salinity stress. Collectively, our genome-wide characterization, evolutionary, and expression analysis suggested that the MsGRAS proteins might play crucial roles in response to abiotic stresses and hormonal cues in alfalfa. For the breeding of alfalfa, it provided important information on stress resistance and functional studies on MsGRAS and hormone signaling.

## 1. Introduction

Transcription factors (TFs) as regulatory proteins could bind to specific DNA sequences (cis-acting elements), which might be located in the promoters of target genes, and play a vital role in plant development, as well as in response to abiotic stresses, such as drought, salt, chilling and heat [1]. The GRAS transcription factors are proposed to be plant-specific regulation proteins, which are supported by their possession of certain structural features [2]. The GRAS family was defined, after GAI (gibberellic acid insensitive), RGA (repressor of GA1–3 mutant), and scarecrow (SCR) were identified as family members. GAI and RGA play a vital role in the gibberellin-dependent signal transduction process, and SCR is involved in regulating the radial tissue differentiation in roots [3,4,5]. Typically, the GRAS protein sequences consist of 400–700 amino acids (aa), including a highly conserved C-terminal and a variable N-terminal. Its C-terminal is composed of five conserved domains, namely SAW, LRI, LRII, PFYRE, and VHIID [2,6]. The N-terminal is the functional component of the GRAS protein, as its IDRs (intrinsically disordered regions) could bind with different other proteins [7].

Recently, the GRAS gene family have received genome-wide characterization in various plant species, such as *Arabidopsis thaliana* [8], rice (*Oryza sativa*) [9], soybean (*Glycine max*) [10], tomato (*Solanum lycopersicum*) [11], pepper (*Capsicum annuum*) [12], *Medicago truncatula* [13], *Camellia sinensis* [14], and tartary buckwheat (*Fagopyrum tataricum*) [15]. According to the conserved motifs and sequence similarity, the GRAS family members in model plants have been separated into eight distinct subfamilies, including DELLA, SCL3 (Scarecrow-like), SCR, SHR (Short-root), LAS (Lateral suppressor), PAT1 (phytochrome A signal transduction 1) [16], HAM (Hairy meristem), and LISCL [9]. The *GRAS* gene families have been identified, and their subfamily classification is different in multiple plants. For example, the Chinese cabbage GRAS family contains 48 genes, which are divided into 8 subfamilies according to *Arabidopsis* classification [17]. The 47 *GRAS* genes in tartary buckwheat were classified into 10 subfamilies: LISCL, HAM, DELLA, SCR, PAT1, SCL4/7, LAS, SHR, SCL3, and DLT [15]. A total of 50 *CaGRAS* members were identified in the pepper genome and divided into 10 subfamilies [12]. There are 150 GRAS proteins in the *Gossypium hirsutum* genome. *G-GRAS*, out of 15 subfamilies, is a unique one in cotton [18]. A total of 116 *GmGRAS* genes could be categorized into 9 subfamilies, with 1 gene of *GmGRAS55* unclassified in *G. max* [10]. Notably, 37 GRAS genes were grouped into up to 17 GRAS subfamilies in *Lagenaria siceraria* [19].

GRAS proteins drew a lot of research attention, especially in 5 major directions, i.e., gibberellic acid (GA) signaling, plant development, and stress responses. Firstly, DELLA proteins, as key GRAS negative regulators, regulate gene expression of the positive regulators in GA signaling, such as GA 20-oxidase, GA receptor, and a transcriptional regulator of SCARECROW-LIKE3 (SCL3), and enable the regulation of GA feedback [20]. GAs could induce the degradation of repressors DELLA proteins, which are encoded by *GAI*, *RGA*, *RGL1*, *RGL2*, and *RGL3* in *Arabidopsis*, with a conserved N-terminal domain named “DELLA” [4,21,22,23]. Secondly, GRAS family members are involved in diverse fundamental processes of plant growth and development. For example, the combination of SHR and SCR proteins forms a SCR/SHR complex, which is involved in the formation of the root radial pattern [3]. LAS protein participates in the growth and development of collateral buds in *Arabidopsis* [24]. The reduced expression level of *GRAS2* (PAT1 subfamily) resulted in the decreased weight in tomatoes [25]. In the *ham* mutant with a loss of *GRAS* family member of HAM in *Petunia hybrida*, shoot meristems differentiate post-embryonically as continuations of the subtending stem [26]. Silencing the *SLFSR* gene in tomato can significantly prolong the shelf-life of fruit, decrease the rate of cell degradation, and reduce the expression level of cell wall modification-related genes [27], while overexpression of *SLGRAS24* inhibits cell division and expansion in the early development of tomato fruit [28]. Thirdly, GRAS proteins are considered to act as key regulators to participate in various abiotic stress. For instance, the *PESCL7* gene of poplar (*Populus euphratica*) was overexpressed in *A. thaliana*, and it showed a stronger tolerance to salt and drought stress, with increased enzyme activity, in transgenic seedlings [29]. The GRAS transcription factor BrLAS from *Brassica rapa* is involved in drought stress tolerance in transgenic *Arabidopsis* [30].

The alfalfa genome assemblies of “Zhongmu No.1”, “XinJiangDaYe”, and “*M. sativa* spp. caerulea voucher” [31,32,33] were released in 2020, and it is more convenient to screen the entire *GRAS* gene family, considering the important role of GRAS proteins in plants and the lack of previous *GRAS* gene family information in alfalfa. In this study, we systematically determined 51 GRAS transcription factors in alfalfa, based on the best assembly of “Zhongmu No.1”, and classified them into 9 major groups. We carried out a comprehensive analysis on phylogenetic construction, conserved motif, chromosomal distribution, gene structure, intron/exon organization, and protein structure. Furthermore, the expression profiles of the GRAS members showed a significant difference under salinity and drought stress treatments. We selected six *MsGRAS* genes to confirm their expression response to abiotic stresses and hormone treatments via qRT-PCR experiment. The results provided a basis for further investigating the roles of MsGRAS proteins and stress breeding in alfalfa.

## 2. Results

### 2.1. Identification and Multiple Sequence Analysis of GRAS Genes in Alfalfa

**Figure 1:**
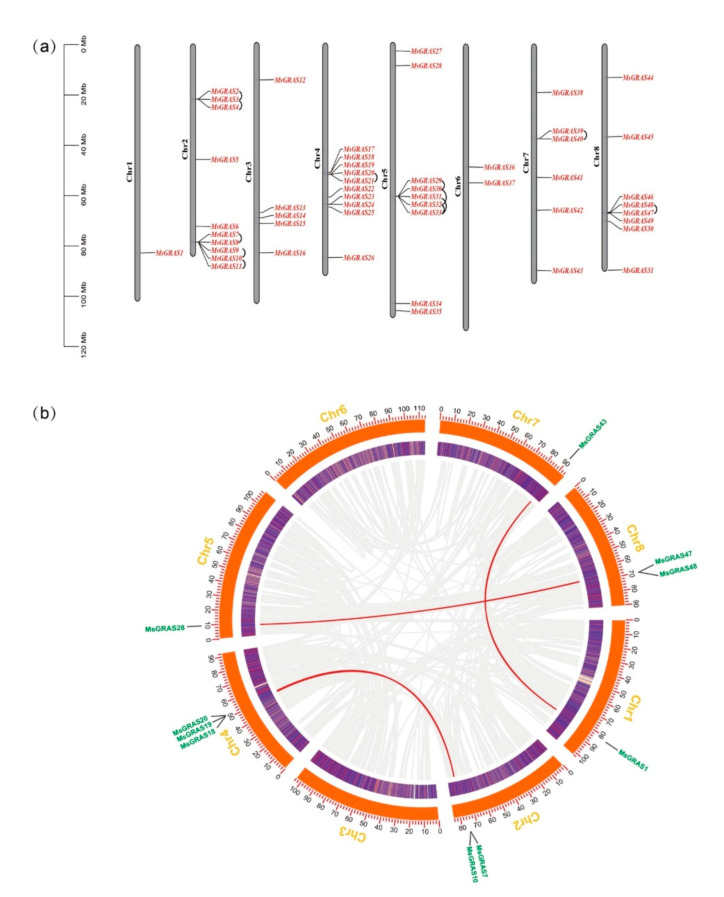
Chromosomal distribution and duplication events of MsGRAS proteins. (**a**) The tandem duplicated genes are marked by black arc trajectory. (**b**) The segmentally duplicated genes are connected by red lines, referring to the ten genes highlighted in green.

We firstly retrieved 32 GRAS proteins (AtGRAS) in *A. thaliana* from the TAIR website (https://www.arabidopsis.org/ accessed on 11 September 2020). In order to obtain the GRAS protein of alfalfa, we used the *Arabidopsis* AtGRAS proteins as a query in a BLASTP search against the *M. sativa* genome of “Zhongmu No.1” [33], which was downloaded from the FIGSHARE database (https://figshare.com/ accessed on 11 September 2020). Subsequently, the online bioinformatics tool of Batch Web CD Search Tool (BWCDST) on the NCBI website (https://www.ncbi.nlm.nih.gov/Structure/bwrpsb/bwrpsb.cgi accessed on 11 September 2020) identified about 51 sequences containing the conserved GRAS domain. The 51 putative MsGRAS proteins were finally determined using the Pfam database (http://pfam.xfam.org/ accessed on 11 September 2020), based on the conserved GRAS domain (PF03514.13), and used for further analysis (Table S1). The 51 alfalfa *GRAS* genes were mapped onto eight alfalfa chromosomes (Chr1–8) and named based on their chromosomal locations (Figure 1a). The alfalfa *GRAS* genes display uneven distributions across the whole eight chromosomes. Chr1 and Chr6 contained one (1.9%, i.e., MsG0180004788.01 (*MsGRAS1*)) and two (3.9%, MsG0680032758.01 (*MsGRAS36*) and MsG0680033030.01 (MsGRAS37)) *MsGRAS* genes, respectively. By contrast, most of the GRAS protein are localized on Chr2 (*n* = 10, 19.6%), Chr3 (*n* = 5, 9.8%), Chr4 (*n* = 10, 19.6%), Chr5 (*n* = 9, 17.6%), Chr7 (*n* = 6, 11.8%), and Chr8 (*n* = 8, 15.7%). In this study, we found that 12 pairs of *GRAS* genes were evidence of tandem duplication events, and clustered into 5 regions on alfalfa Chr2 (*MsRAS2/3*, *MsRAS3/4*, *MsRAS7/8, MsRAS8/9*, *MsRAS10/11*), Chr4 (*MsRAS20/21*), Chr5 (*MsRAS29/30*, *MsRAS30/32*, *MsRAS31/33*, *MsRAS32/33*), Chr7 (*MsRAS39/40*), and Chr8 (*MsRAS47/48*). In addition to tandem duplication events, three segmental duplication events were also identified for ten *MsGRAS* genes, i.e., *MsGRAS1* vs. *MsGRAS43*, *MsGRAS7/10* vs. *MsGRAS18/19/20*, and *MsGRAS28* vs. *MsGRAS47/48*, which are located on chromosomes of 1, 2, 4, 5, 7, and 8 (Figure 1b).

**Figure 2:**
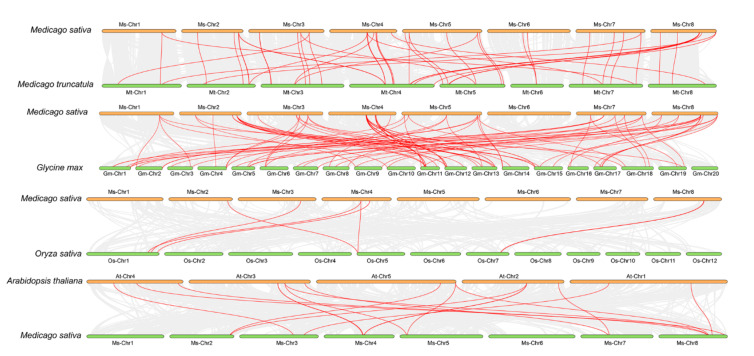
Syntenic analysis of *MsGRAS* genes in *M. sativa* in comparison with those in four plant species (*M. truncatula*, *G. max*, *O. sativa*, and *A. thaliana*).

We carried out a collinearity analysis on *MsGRAS* genes with those in a monocotyledonous plant of rice (*Oryza sativa*) and three dicotyledonous plants of *Arabidopsis*, soybean, and *M. truncatula* (Figure 2), to further understand the collinearity of the *MsGRAS* genes. *MsGRAS* genes displayed various numbers of syntenic lines with four species. A total of 36 *MsGRAS* genes showed a syntenic relationship with those in soybean, and 28 corresponding orthologs were identified between *M. sativa* and *M. truncatula*. Meanwhile, only 6 and 12 *MsGRAS* genes showed syntenic relationships with those in *O. sativa* and *A. thaliana*, respectively (Figure 2 and Table S2).

Sequence analyses revealed that the size of the MsGRAS proteins vary greatly in length, from 98 aa (MsGRAS24) to 1002 aa (MsGRAS12). The proteins molecular weight ranged from 23.44 kD (MsGRAS49) to 112.7 kD (MsGRAS12), and the isoelectric point (pI) ranged from 4.77 (MsGRAS45) to 9.24 (MsGRAS15). Subcellular localization prediction by WoLF PSORT tool showed that 33 MsGRAS proteins are located in the nucleus, 12 in the cytoplasmic, and 6 in the chloroplast (Table 1), while TargetP prediction results were all “Other”, rather than chloroplast or mitochondria. The results showed that MsGRAS protein might function in diverse microcellular environments.

**Figure 3:**
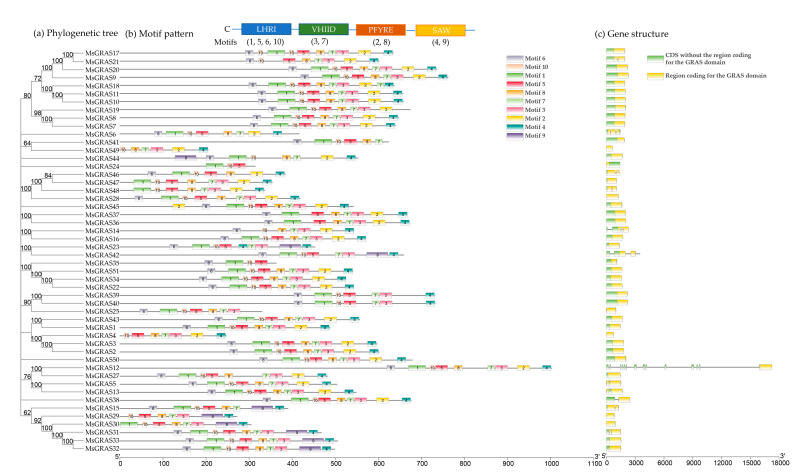
Phylogenetic relationship, motif pattern, and gene structure of *MsGRAS* genes. (**a**) Phylogenetic tree of *MsGRAS* was constructed based on the full-length sequences of proteins using MEGA-X [34] based on the neighbor-joining (NJ) method. Bootstrap values for 1000 replicates were shown above the lines. (**b**) The motifs of *M. sativa* GRAS proteins predicted by MEME and plotted in TBtools. The motifs of 1–10 are displayed in different colored boxes. The protein length can be estimated using the scale at the bottom. The schematic of four conservative domains at the C-terminal regions of the MsGRAS members was combinations of motifs. (**c**) Gene structure of *MsGRAS* genes. Region coding for the GRAS domain (yellow) was determined in Batch Web CD Search Tool (BWCDST) on the NCBI website and plotted in TBtools software. “CDS without the region coding for the GRAS domain” (green), where the coding region does not cover the GRAS domain, are plotted based on the alfalfa genome annotation. gff file, which was input into TBtools together with hit export from BWCDST.

**Figure 4:**
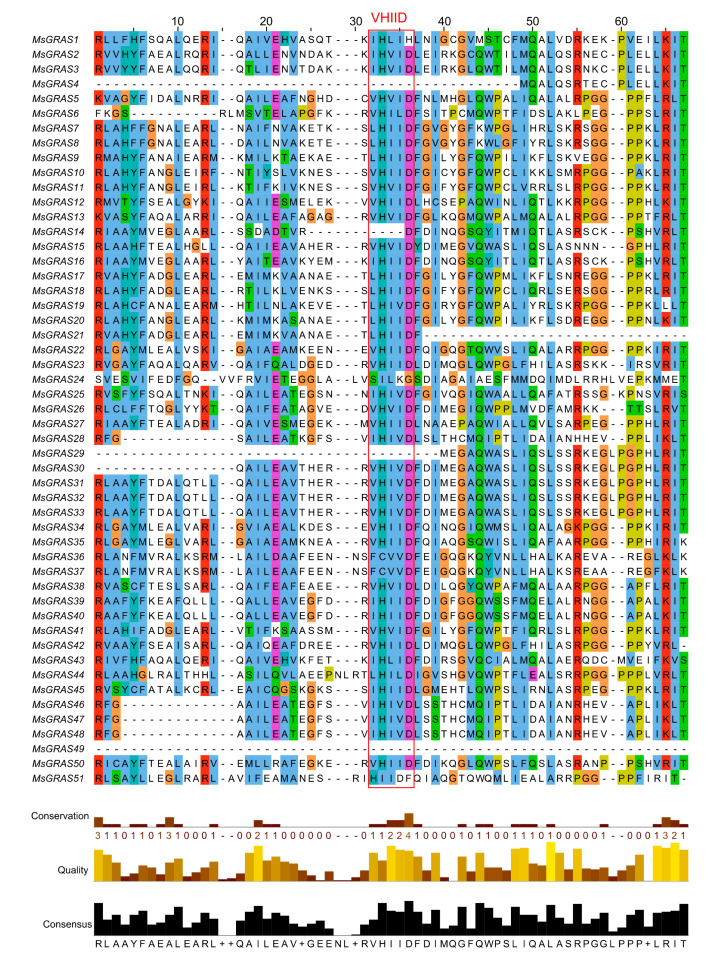
VHIID domain of 51 MsGRAS proteins based on sequence alignment. The most conserved GRAS domain of VHIID was boxed in red.

The online MEME search tool was used to further look into the conversation of MsGRAS proteins, and 10 distinct conserved motifs (named as motif 1–10) were found (Figure 3a,b and Table S3). Based on the alignment of predicted GRAS domain sequences, we found that most of the MsGRAS proteins (94%) contained motifs of 4, 6, and 7, and 2 copies of motif 7 were separated by motifs of 2 and 3 in MsGRAS18. Four protein pairs of MsGRAS2/3, MsGRAS7/8, MsGRAS10/11, and MsGRAS39/40 shared identical motif patterns, and MsGRAS24 held only three motifs of 1, 5, and 10. Multiple sequence alignments showed that most of the MsGRAS proteins possess the N-terminal and the highly conserved C-terminal, and the conserved domains located in the C-terminal included VHIID, LHRI, and SAW. Among the whole MsGRAS proteins, 47 MsGRAS contain VHIID and LHRI domains, and 49 proteins contain SAW domains (Figure 4 and Figures S1–S3). The motifs were located in the four GRAS domains, including LHRI (motif1, motif5, motif6, motif10), VHIID (motif3, motif7), PFYRE (motif2, motif8), and SAW (motif4, motif9), and shared across almost all MsGRAS members. We further found that MsGRAS24 with the gene ID of MsG0480021771.01.T01 is wrongly annotated in the alfalfa genome reference of “Zhongmu No.1”. Based on our unpublished PacBio sequencing dataset for a pool of root, stem, leaf, etc., in alfalfa “Zhongmu No.1”, one full-length transcript was retrieved for this gene (Table S1), and based on the genomic coordinates of this transcript, its gene annotation and structure were updated without long introns (Figure 3c).

### 2.2. Phylogenetic Analysis and Classification of GRAS Members

**Figure 5:**
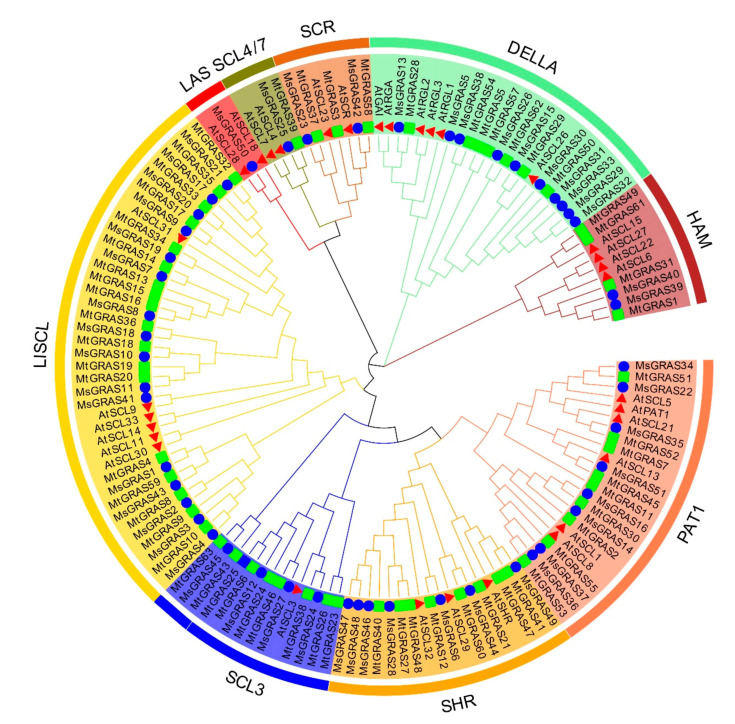
Phylogenetic analysis of GRAS proteins. The phylogenetic tree was generated by the neighbor-joining method derived from Clustal X alignment of GRAS amino acid sequences from *M. truncatula*, *Arabidopsis*, and *M. sativa*, respectively. Protein IDs are marked in colors for the three species, i.e., *M. truncatula* in green, *Arabidopsis* in red, and *M. sativa* in blue.

To fully uncover the evolutionary history of the *GRAS* gene family in alfalfa, 51 *MsGRAS* genes were compared with 32 and 59 *GRAS* genes in *Arabidopsis* (https://www.arabidopsis.org/index.jsp accessed on 5 October 2020) and *M. truncatula* (http://www.medicagohapmap.org/ accessed on 5 October 2020), respectively, and an unrooted phylogenetic tree was constructed using the neighbor-joining method in MEGAX. Based on the phylogenetic tree (Figure 5), all 51 MsGRAS proteins could be grouped into 9 subfamilies (SCL3, HAM, DELLA, SCR, LAS, LISCL, SHR, PAT1, and SCL4/7), which is consistent with the previous studies on *A. thaliana* [9] and *M. truncatula* [13]. A total of 4, 2, 10, 2, 1, 16, 7, 8, and 1 *MsGRAS* genes corresponded to subfamilies of SCL3, HAM, DELLA, SCR, LAS, LISCL, SHR, PAT1, and SCL4/7, respectively (Figure 5). The LISCL subfamily herein contained 16 members and was the largest *MsGRAS* gene subfamily in this study.

### 2.3. Transcriptome Analysis of the Alfalfa Leaves under Abiotic Stress

**Figure 6:**
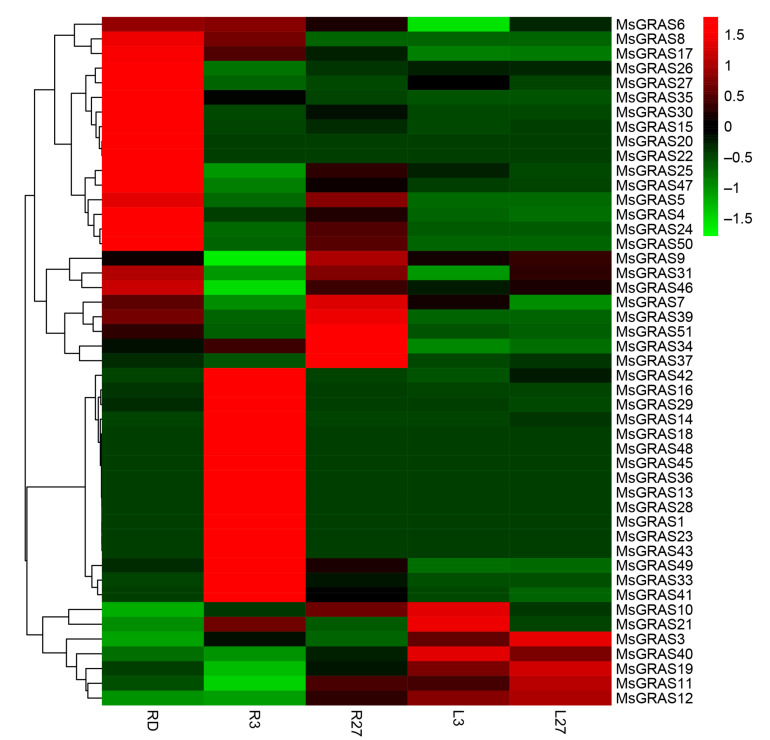
Gene expression profiles of *GRAS* members in *M. sativa*. R3/L3/27: The roots (R) or leaves (L) were treated for 3 or 27 h under salinity stress; RD: Roots under drought stress. The color scale represents log10 expression values.

To investigate the expression profiles of *MsGRAS* members under abiotic stresses (salt and drought), we downloaded publicly available leaf and root transcriptome data from NCBI (Bioproject PRJNA657410 and PRJNA525327) [35] and conducted RNA-seq analysis. The alfalfa plant was treated with salt stress for 3 (L3 for leaf and R3 for root) and 27 h (L27 for leaf and R27 for root) with the control (L0 for leaf and R0 for root), while drought stress was also applied for roots under drought stress (RD). In root tissue, 35 *MsGRAS* genes were expressed in R0 (Table S4), and the expression of 4 and 5 *MsGRAS* genes changed significantly in R3 and R27, respectively, including *MsGRAS51/13*. In leaf tissue, 29 *MsGRAS* genes were expressed in L0 (Table S4), and the expression levels of 17 and 19 *MsGRAS* genes increased in L3 and L27, respectively (Figure 6, Table S4). Actually, 14 *MsGRAS* genes were not expressed in both root and leaf. Under the drought stress, 13 *MsGRAS* genes were insensitive, and about 41% of *MsGRAS* genes showed significant differences, of which eight genes belonged to the PAT1 subgroup.

**Figure 7:**
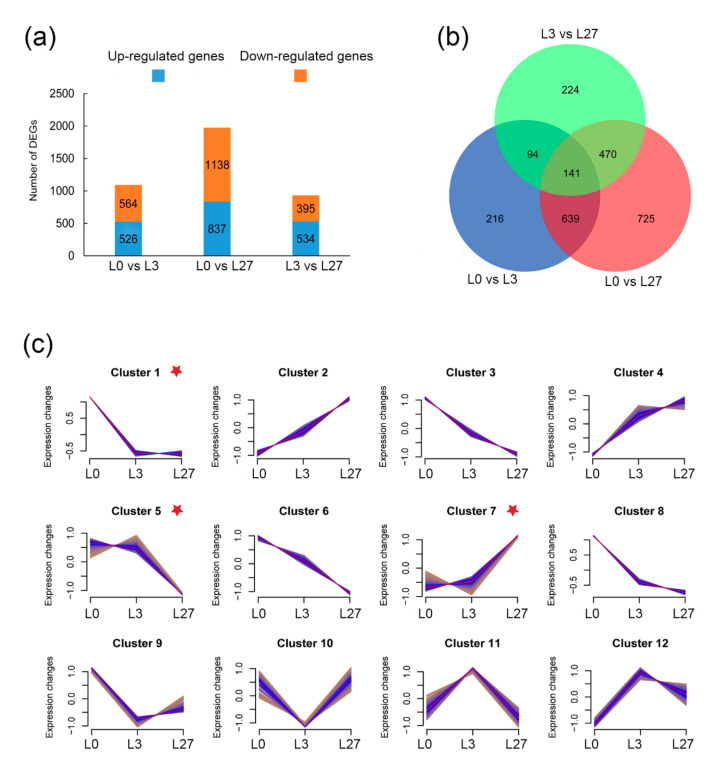
Analysis of differential expression and cluster analysis under salt stress in *M. sativa*. (**a**) Comparisons of DEGs in alfalfa leaf tissue in response to salinity stress. The blue means up-regulation, orange means down-regulation. (**b**) Venn diagram of DEGs in three comparisons. L0 is the control group of leaf and L3 and L27 indicate salinity treatments on a leaf for 3 and 27 h, respectively. (**c**) Clustering of DEGs. A total of 12 clusters were achieved, and 6 *MsGRAS* genes were found in three ones, labeled with a red star.

Furthermore, we performed a differential expression analysis of all genes in alfalfa leaves under salinity stress compared with the control groups. A set of differentially expressed genes (DEGs) for each salinity stress treatment were determined according to their significance with a cutoff of *p*-value < 0.05 and |log2(fold change)| > 1 (Table S5 and Figure 7a). The result showed that a total of 1090, 1975, and 929 DEGs, in response to NaCl treatments, were detected in the three comparisons (L0 vs. L3, L0 vs. L27, L3 vs. L27), respectively. The DEGs with down-regulated ones in L0 vs. L27 were significantly more than those in L0 vs. L3. Compared to the control, there were 526 up-regulated and 564 down-regulated DEGs under 3 h salinity stress, whereas 837 up-regulated and 1138 down-regulated DEGs in response to 27 h NaCl treatment in alfalfa leaves. A total of 929 DEGs were detected in L3 vs. L27, including 534 up-regulated and 395 down-regulated ones. The Venn diagram further showed that 141 DEGs were common in the three comparisons (Figure 7b). These results suggested that the alfalfa plant was more sensitive to salinity stress for a longer time, e.g., 27 h in this case, with more DEGs.

To trace the partners of *MsGRAS* genes in salinity response, we analyzed the co-expression of these DEGs and divided them into 12 clusters. A total six *MsGRAS* genes were placed in three clusters, i.e., *MsGRAS37* in cluster 1; *MsGRAS*5, *MsGRAS13*, and *MsGRAS51* in cluster 5; *MsGRAS27* and *MsGRAS50* in cluster 7 (Figure 7c). In cluster 1, the gene expression decreased rapidly at 3 h of salt stress treatment and maintained at 27 h. The gene expression in cluster 5 did not change significantly at 3 h and showed an obvious down-regulation trend at 27 h, while the gene expression pattern in cluster 7 is up at 27 h.

**Figure 8:**
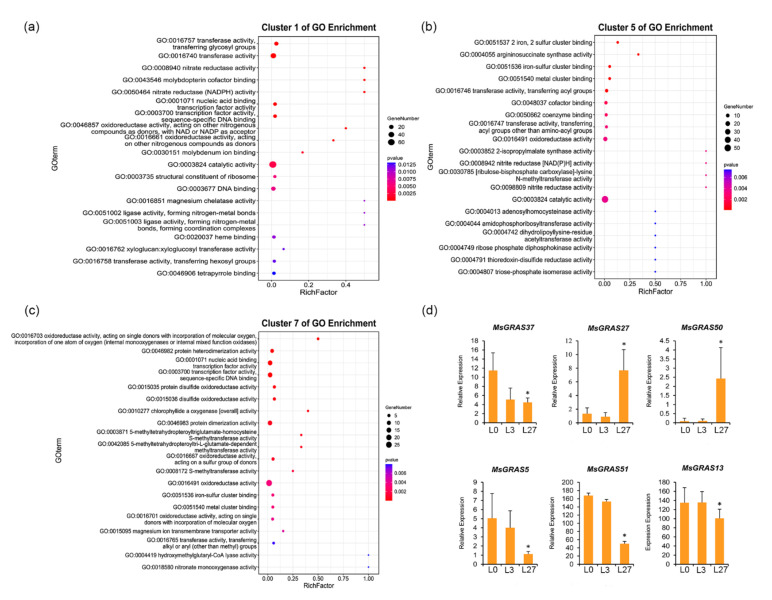
GO enrichment analysis of DEGs shared by clusters of 1, 5, 7 and the relative expression of *MsGRAS* genes of #5, 13, 27, 37, 50, 51. (**a**) Cluster 1 of GO enrichment. (**b**) Cluster 5 of GO enrichment. (**c**) Cluster 7 of GO enrichment. (**d**) Expression profiles of *MsGRAS* genes of #5, 13, 27, 37, 50, 51 from RNA-seq data (* *p* < 0.05, Student’s *t*-test).

To identify functions of salinity-responsive partners of *MsGRAS* genes in alfalfa, we conducted GO term enrichment analysis on DEGs in cluster 1, cluster 5, and cluster 7 in leaf. In the GO enrichment graph, the *p*-value is sorted from small to large to indicate the significance of the enrichment degree (Figure 8a). The GO enrichment results showed that the majority of DEGs in cluster 1 were involved in molecular function categories, including ‘catalytic activity’, ‘transferase activity’, ‘transferase activity transferring glycosyl groups’, and ‘DNA binding’. The DEGs in cluster 5 were involved mostly in molecular functions, including ‘catalytic activity’, ‘oxidoreductase activity’, ‘cofactor binding’, and ‘transferase activity’. Those in cluster 7 were mainly enriched in terms of ‘lysine methyltransferase activity’, ‘transcription factor activity’, and ‘protein heterodimerization activity’. The expression of *MsGRAS*5, *MsGRAS*13, *MsGRAS*37, and *MsGRAS*51, which belong to the DELLA and PAT1 subfamilies, decreased significantly at 27 h (Figure 8d), which indicated the role of DELLA and PAT1 proteins in response to salt stress.

### 2.4. Validation of Expression Profiles of MsGRAS Genes under Abiotic Stress and Hormone Treatments

**Figure 9:**
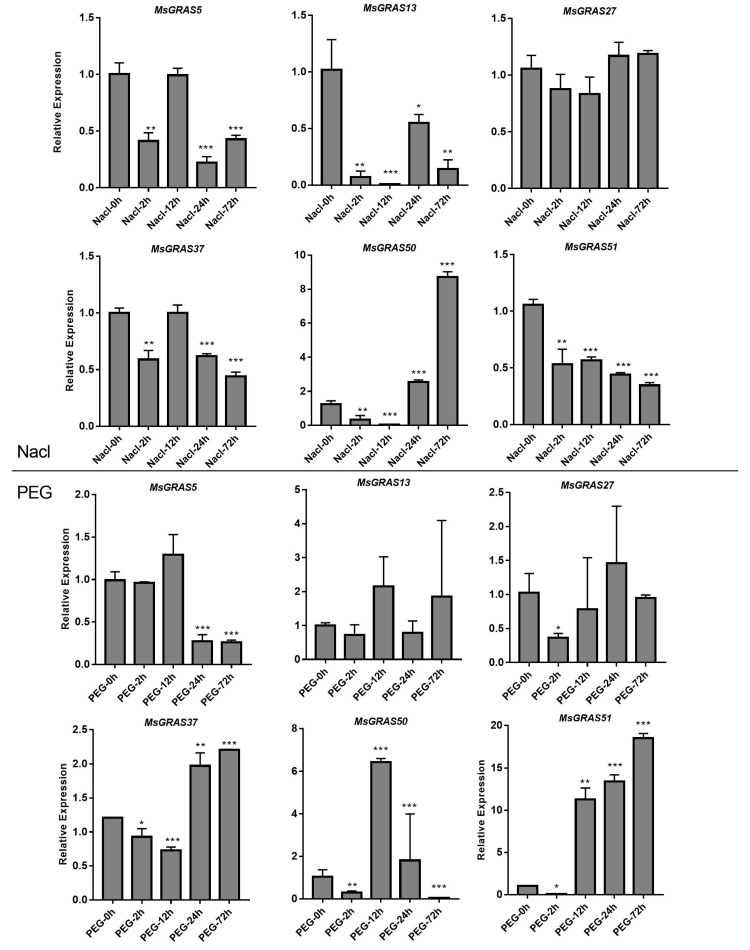
Expression profiles of six selected *MsGRAS* genes in response to salt and drought stress treatments. Expression levels are normalized to Ms-ACTIN, and error bars indicate standard deviation among three biological replicates. Asterisks indicate the corresponding genes were significantly up-regulated or down-regulated compared with the control (* *p* < 0.05, ** *p* < 0.01, *** *p* < 0.001; Student’s *t*-test).

Among all 51 *MsGRAS* genes, expressions of 6 (*MsGRAS*5, *MsGRAS*13, *MsGRAS*27, *MsGRAS*37, *MsGRAS*50, and *MsGRAS*51) were highly induced by salinity and drought stresses. We designed primers for the six selected genes and conducted qRT-PCR experiments in leaf tissues (Table S6). The gene expression profiles of the six selected genes were generally consistent with the RNA-seq data (Figure 8d and Table S7). The results showed that upon salinity treatment, *MsGRAS50* expression was significantly induced (*p* < 0.01), while the other four *MsGRAS* genes’ expression was down-regulated significantly. Under drought stress, the expression of *MsGRAS51* increased gradually. After 72 h of treatment, *MsGRAS51* expression was about 20 times that of the control, implying its putative roles in stress tolerance. Interestingly, the expression of three genes (*MsGRAS5, MsGRAS13,* and *MsGRAS50*) were only up-regulated at 12 h. However, *MsGRAS27* showed no significant influence in both salt and drought treatment (Figure 9).

**Figure 10:**
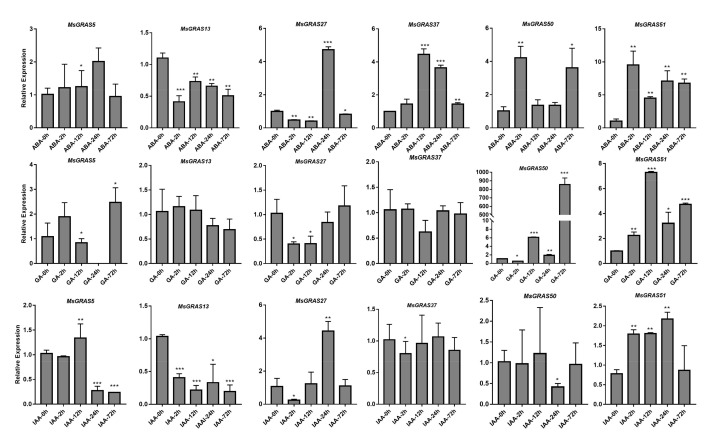
Expression of *MsGRAS* genes in response to three hormones of ABA, GA, and IAA. Expression levels are normalized to Ms-ACTIN, and error bars indicate standard deviation among three biological replicates. Asterisks indicate the corresponding genes were significantly up-regulated or down-regulated compared with the control (* *p* < 0.05, ** *p* < 0.01, *** *p* < 0.001; Student’s *t*-test).

Previous studies have demonstrated that GRAS genes are key regulators of germination [21], seedling growth [2], lateral root development, and shoot meristem maintenance [6]. Plant hormones have been widely reported for their roles in the regulation of various aspects of plant growth. Upon drought and salinity stress, gene expression profiles showed that *MsGRAS* genes play a vital role in response to abiotic stress. We further verified the expression profiles of 6 selected *MsGRAS* genes in alfalfa seedlings under exogenous ABA, GA, and IAA treatments by qRT-PCR. We found that the hormone treatments resulted in a wide variety of *MsGRAS* gene expression profiles (Figure 10, Table S8). It showed that *MsGRAS5* was not responsive to ABA, and the other five genes’ expression, with one up- and four down-regulated, was significantly different from the control. Four *MsGRAS* genes showed significantly different expressions after treatments of both GA and IAA. The up-regulated expression levels of *MsGRAS51* responded to all three hormones. It suggested that the *MsGRAS* TFs played vital roles in response to ABA, GA, and IAA stress signaling. Under GA treatment for 72 h, *MsGRAS50* was significantly up-regulated, with >800 folds of the control. *MsGRAS5* as DELLA protein showed a significant response to gibberellin treatment and no expression at all at 24 h. The expression levels of six *MsGRAS* genes varied obviously in response to hormone treatments, suggesting that the *MsGRAS* genes have been involved in different pathways in alfalfa seedlings.

## 3. Discussion

The plant-specific GRAS proteins are involved in the regulation of plants developmental and physiological processes, such as seed germination, fruit ripening, and various biotic and abiotic stresses [27,28,29,36]. To date, several attempts have been undertaken to group members of the GRAS family into subfamilies that reflect their evolutionary history in a large number of plant species, such as *A. thaliana* [9], *M. truncatula* [13], *G. max* [10], tomato [11], Chinese cabbage [17], *C. annuum* [12], and *G. hirsutum* [18]. In contrast, alfalfa, as a member of the forage crops, is one of the most important hay and silage productions worldwide. It is difficult to screen the GRAS family members in alfalfa, as alfalfa is an autotetraploid, open-pollinated legume with a high level of heterozygosity in the genome [37,38]. A total of 51 GRAS genes in alfalfa were more than those in *Arabidopsis* (32) [9], *Chrysanthemum morifolium* (23) [39], *p. mume* (45) [40], castor bean (46) [41], cabbage (48) [17], similar to those in pepper (50) [12] and tomato (53) [11], but less than those in Populus (106) [42], soybean (117) [10], and *G. hirsutum* (150) [18]. *MsGRAS* gene members are divided into nine subfamilies, including SCL4/7 with *MsGRAS35* and *MtGRAS39* (Figure 5). However, in *M. truncatula*, *MtGRAS39* was classified into the LAS subfamily rather than SCL4/7. It is noted that our subfamilies in alfalfa are the same as those in soybean [10] and *Arabidopsis* [9].

### 3.1. Duplication of MsGRAS Genes

In this investigation, the identified *MsGRAS* genes are unevenly distributed on eight chromosomes of the alfalfa genome. Notably, Chr2 and Chr4 contained most of *MsGRAS* genes (20 genes), which mainly originated from all of the nine subfamilies, and Chr1 is the “cold” region, including only one *MsGRAS* gene (Figure 1a). In *M. truncatula*, Chr2 and Chr4 are also the “hot” regions, with 15 *MtGRAS* genes [13]. Furthermore, based on the distribution of *MsGRAS* in chromosomes, we discovered the gene duplication events. Gene loss and replication events greatly promote the evolution of species, providing raw materials for generating novel gene functions and expanding gene families [43]. Most plants have experienced one or more rounds of ancient polyploidy, such as *Arabidopsis*, which had undergone two recent whole-genome duplications [44]. It is speculated that gene replication may be one of the main factors for the number variation of GRAS family members. We discovered 12 pairs of *MsGRAS* genes as evidence of tandem duplication events on five chromosomes (Chr2, Chr4, Chr5, Chr7, and Chr8), among which six belonged to the LISCL subfamily, and three segmental duplication events for ten *MsGRAS* genes (Figure 1b). It seems that tandem duplication contributed more to *M. sativa* GRAS expansion than segmental duplication. For example, LISCL subfamily expansion in alfalfa is also found in *M. truncatula* [13]. Segmental duplication and tandem duplication are considered to represent principal evolutionary patterns in plants. Segmental duplications are produced by polyploidy and chromosomal rearrangement because of numerous duplicated chromosomal blocks in plant genomes [45]. Interestingly, almost all genes in MsGRAS families have no introns. In the *GRAS* gene family, the higher percentage of intronless genes, the closer the evolutionary relationship of the GRAS members [46]. Intron deficiency is common in other large gene families, for example, DEAD-box RNA helicases, small auxin-up RNAs (SAUR) gene family, and the F-box transcription factor family [47,48,49]. Intronless genes are also common in prokaryote genomes, but there are three explanations for the appearance of intronless genes in eukaryote genomes, i.e., horizontal gene transfer, intronless gene reverse transcription, and intronless gene replication [46]. In addition, the GRAS domain proteins were discovered in bacteria [50], and thus, horizontal gene transfer from ancient prokaryote genomes might be a possibility to explain the abundant intronless genes in the plant GRAS gene family.

### 3.2. Expression and Function of MsGRAS Genes

The *GRAS* members in the same subfamily commonly exhibit functional similarities. Structural analysis provides a clue to locate which subgroup of *GRAS* is of ancient origin [51]. All of the ten predicted alfalfa DELLA proteins (MsGRAS5, MsGRAS13, MsGRAS15, MsGRAS26, MsGRAS29, MsGRAS30, MsGRAS31, MsGRAS32, MsGRAS33, and MsGRAS38) were classified into the DELLA subgroup, together with the *Arabidopsis* DELLA proteins of AtGAI, AtRGA, AtRGL1, AtRGL2, and AtRGL3. In *Arabidopsis*, the DELLA family consists of GAI, RGA, RGL1, RGL2, and RGL3 [4,21,23,52]. To date, the functions of the AtGAI and AtRGA proteins have been clearly illuminated in *Arabidopsis*, and DELLA protein, as a putative transcriptional regulator, is a subbranch of the GRAS gene family, playing a negative regulatory role in gibberellin (GA) signal transduction [53]. DELLA proteins restrain the development of plants, whereas GA relieves plants of DELLA-mediated growth restraint [54,55]. After GA treatment, the expression of *MsGRAS5* significantly changed, especially without expression at 24 h, indicating its potential role in gibberellin regulation in alfalfa.

It is known that GRAS proteins might interact with each other in a pathway. The GRAS family transcription factors of SCR and SHR are involved in root tissue formation, and the two subfamilies AtSHR and AtSCR, were considered to function importantly in maintaining stem and root cell meristem [12,56]. As a positive regulator, SCL3 acts to integrate and maintain a functional GA pathway by eliminating the negative regulation of DELLA to control the dynamic balance of GA in root development for cell elongation [57]. In this study, the SCL3 subfamily contained four MsGRAS proteins (MsGRAS12, 24, 27, and 45), which is implying a similar function in root development. In addition, LISCLs are of the largest GRAS subfamily in alfalfa and involved in the regulation of microsporogenesis in *Lilium longiflorum* [43], while LAS proteins act as branching regulators, which is required to establish axillary meristems at a distance to the SAM during vegetative development [24,58,59].

### 3.3. Possible Functions of MsGRAS Members

The gene expression profiles could provide key clues to predict their possible functions. As a broad-spectrum phytohormone, ABA is involved not only in regulating plant growth and development but also in coordinating in many aspects of stress signal transduction pathways during abiotic stresses. ABA mediates many stress responses and activation of genes involved in tolerance adjustment [60]. When plants are subjected to biotic and abiotic stress, cells are prone to oxidative stress, which is due to the increase of the content of reactive oxygen species (ROS) [61]. The phytohormone GA is known to play an important role in regulating a diverse array of developmental processes, such as seed development and germination, organ elongation, and control of flowering time. The GRAS subfamily has been reported to function as repressors of gibberellin (GA) signaling [2]. A previous study reported that DELLA proteins thus repressed ROS accumulation, interrupts ROS-induced cell death, and hence enhance plant biotic and abiotic stress tolerance [62].

Recent studies have shown that *GRAS* genes have multiple functions in plant abiotic stress tolerance. The rice GRAS transcription factor gene *OsGRAS23* was induced by osmotic stress, and overexpression of this gene enhanced the drought resistance in transgenic rice plants by regulating the expression of stress-responsive genes [63]. In *M. sativa*, *MsGRAS37* and *MsGRAS51* showed a high expression level under PEG treatment. Moreover, the expression levels of several *MsGRAS* genes (e.g., *MsGRAS5/13/50/51*) were dramatically regulated by salinity stresses. It suggested they played critical roles in the abiotic stress tolerance in *M. sativa*. In our study, the expression of *MsGRAS51* significantly responded to all the treatments of hormone and stress, indicating a potential regulatory role in alfalfa development and stress resistance.

## 4. Materials and Methods

### 4.1. Plant Materials

In this study, the seeds of *M. sativa* cultivar Zhongmu No.1 were harvested in the autumn of 2019 at the Gansu Forage and Pasture Research Station of the China Agricultural University (attitude 39°37′ N, longitude 98°30′ E; elevation 1480 m). *M. sativa* seeds were placed on moistened filter paper in dishes and then sustained in a growth chamber with 16 h light/8 h dark at 20 °C constant temperature. Seven-day-old seedlings were then subjected to different experiments. For each experiment, all samples were immediately frozen in liquid nitrogen and stored at −80 °C for RNA extraction.

For the expression analyses of *GRAS* genes’ response to different treatments, the seven-day-old seedlings were transferred into the flasks with 1/2 MS liquid medium and grew in a controlled growth chamber under 16/8 h light/dark regime at 20 °C. After the third leaf was fully expanded, the plants were supplied with 1 mmol·L^−1^ IAA, 1 mmol·L^−1^ GA3, 1 mmol·L^−1^ ABA, 15% PEG6000, and 300 mmol·L^−1^ NaCl to the 1/2 MS liquid medium, respectively. For each treatment, the whole plant was collected at 0, 2, 24, and 72 h with a triplicate.

### 4.2. RNA Extraction and Gene Expression Analysis

The total plant RNAs were extracted using the HUA YUE YANG total RNA Extraction kit (Beijing, China), according to the manufacturer’s instructions. RNA was used as a template for the synthesis of first-strand cDNA. Complementary DNA was generated with reverse transcriptase (TransGen Biotech, Beijing, China) at 42 °C for 15 min, 85 °C for 5 s. Quantitative real-time PCR (qRT-PCR) was conducted according to the instructions of 2 × RealStar Green Fast Mixture (GeneStar, Beijing, China) on CFX96 Touch^TM^ RT-PCR system (Biorad, Los Angeles, CA, USA). The gene-specific primer sequences for qRT-PCR determination are provided in Table S6. Ms-Actin was used as the internal control. Three technical repetitions for each sample were performed, and the relative expression data were calculated according to the 2^−ΔΔCT^ method. Student *t*-test was performed by Graphpad Prism 7 (https://www.graphpad.com/scientific software/prism/ accessed on 5 April 2021). All the error bars were standard deviations (SD) from three biological replicates.

### 4.3. Identification of GRAS Genes in M. sativa

The *M. sativa* “Zhongmu No.1” genome information [31] was downloaded from the figshare website (https://figshare.com/articles/dataset/Medicago_sativa_genome_and_annotation_files/ accessed on 11 September 2020). The 32 GRAS protein sequences of *Arabidopsis* retrieved from the TAIR (https://www.arabidopsis.org/ accessed on 11 September 2020) were used as a query to obtain the possible GRAS proteins in *M. sativa* genome by BlastP search with a cutoff E-value of 1.0 × 10^−10^. Furthermore, the GRAS protein sequences of *M. sativa* were aligned using the HMM model of the HMMER3.0 (http://hmmer.org/ accessed on 11 September 2020) (National Institutes of Health, Bethesda, MD, USA), with an E-value of 0.001 and the removal of redundant sequences. The Hidden Markov Model (HMM) profile (PF03514.13) was used to identify the putative GRAS domain in the Pfam database (http://pfam.xfam.org/ accessed on 11 September 2020) [64].

#### 4.3.1. Chromosomal Distribution and Gene Duplication Events Analysis

The chromosomal locations of 51 GRAS genes were obtained from the *M. sativa* genomic annotation file GFF3 (general feature format). We draw the chromosomal distribution image of *MsGRAS* genes through the TBtools software [65]. The 51 putative GRAS genes were renamed according to their chromosomal locations. The detection and identification of gene duplication events in *MsGRAS* genes were performed using multiple collinear scanning toolkits (MCScanX) [66] with an E-value set to 10^−5^.

#### 4.3.2. Sequence Analyses and Structural Characterization of MsGRAS Genes

The tools from the ExPASy website (https://web.expasy.org/protparam/ accessed on 11 September 2020) (SIB Swiss Institute of Bioinformatics) were used to analyze the basic physical and chemical characteristics, such as sequence length, molecular weight, and isoelectric point of the identified GRAS proteins. The subcellular localization of MsGRAS was predicted on the WoLF PSORT (http://wolfpsort.org accessed on 10 July 2021). The exon-intron structures of *MsGRAS* genes were drawn based on the CDS and the corresponding full-length sequence by using TBtools software [65]. The conserved motifs in MsGRAS proteins were analyzed through the MEME software [67]. The parameters were set as follows: motif width as 15–50 amino acids (aa) and the number of motifs as 10.

### 4.4. Phylogenetic and Collinearity Analysis of MsGRAS Genes

We downloaded the GRAS proteins of *Arabidopsis* and *M. truncatula* from the phytozome (https://phytozome.jgi.doe.gov/pz/portal.html accessed on 5 October 2020), which were used for the phylogenetic analysis of GRAS proteins in plants. Multiple sequence alignments of the 142 *GRAS* genes were performed using the ClustalX software (https://clustalx.software.informer.com/2.1/# version 2.1, accessed on 5 October 2020) with default parameters. The phylogenetic tree based on the alignment was constructed using MEGA-X [34] by neighbor-joining (NJ) method, and the reliability of the obtained trees was assessed with a bootstrap value of 1000. The phylogenetic tree was visualized and illustrated using the online tool EvolView [68] (http://www.evolgenius.info/evolview/# version 3.0, accessed on 5 October 2020). The syntenic relationship between *MsGRAS* genes and *GRAS* genes from *Arabidopsis*, rice, *M. truncatula*, and soybean were determined by using Dual Synteny Plotter (Plant Genome Mapping Laboratory, University of Georgia, Athens, GA, USA, http://chibba.pgml.uga.edu/mcscan2/# version 2.0, accessed on 5 October 2020) software and was visualized by TBtools software (HuaZhong Agricultural University, Wuhan, China).

## 5. Conclusions

We screened 51 *MsGRAS* genes in the alfalfa genome of “Zhongmu No.1” and grouped them into nine subfamilies, which all shared the conversed GRAS domain. We also identified the conserved domain patterns and phylogenetic relationships of these MsGRAS proteins. Our current systematic studies provided gene expression atlas for *MsGRAS* genes. These results provide comprehensive information towards better understanding the molecular basis and impact of *GRAS* gene families on the response of alfalfa to treatments of hormones and stress. Considering that the detailed regulatory mechanisms underlining *GRAS* genes are still not clear in *M. sativa,* it will be of great interest to elucidate individual *MsGRAS* genes in the near future. For instance, *MsGRAS51* might contribute to abiotic stress tolerance in *M. sativa*, and *MsGRAS50* may play an important role in the gibberellin signal transduction pathway. Nevertheless, it provided important information about the GRAS family and a framework for breeding on stress-resistance and hormone signaling transduction in alfalfa.

## Figures and Tables

**Table 1:** Properties and predicted locations of MsGRAS proteins.

Gene Name	Gene Id in “Zhongmu No.1” Assembly	Isoelectric Point	Molecular Weight (Da)	Amino Acids	Subcellular Localization	Expressed in Root	Expressed in Leaf
MsGRAS1	MsG0180004788.01.T01	6.41	55,671.46	487	Nucleus	No	No
MsGRAS2	MsG0280007861.01.T01	5.14	68,709.99	601	Nucleus	Yes	No
MsGRAS3	MsG0280007862.01.T01	5.16	68,262.82	596	Nucleus	Yes	No
MsGRAS4	MsG0280007863.01.T01	5.37	27,925.1	246	Nucleus	No	No
MsGRAS5	MsG0280009128.01.T01	5.47	56,013.31	503	Cytoplasmic	Yes	Yes
MsGRAS6	MsG0280010628.01.T01	5.96	46,813.87	415	Cytoplasmic	Yes	No
MsGRAS7	MsG0280011038.01.T01	5.42	73,172.09	639	Nucleus	No	Yes
MsGRAS8	MsG0280011039.01.T01	5.14	73,950.9	646	Nucleus	Yes	Yes
MsGRAS9	MsG0280011040.01.T01	5.47	86,165.78	761	Nucleus	Yes	Yes
MsGRAS10	MsG0280011041.01.T01	5.26	74,730.24	657	Nucleus	Yes	Yes
MsGRAS11	MsG0280011042.01.T01	5.51	74,363.63	656	Nucleus	Yes	No
MsGRAS12	MsG0380012325.01.T01	6.16	112,665.07	1002	Nucleus	No	No
MsGRAS13	MsG0380015272.01.T01	5	60,086.79	548	Cytoplasmic	Yes	Yes
MsGRAS14	MsG0380015408.01.T01	4.93	61,268.48	542	Nucleus	Yes	Yes
MsGRAS15	MsG0380015569.01.T01	9.24	42,996.71	389	Nucleus	Yes	Yes
MsGRAS16	MsG0380016451.01.T01	5.09	64,121.06	570	Nucleus	Yes	Yes
MsGRAS17	MsG0480020975.01.T01	5.9	72,561.93	634	Nucleus	Yes	Yes
MsGRAS18	MsG0480020976.01.T01	5.99	71,593.66	636	Nucleus	Yes	Yes
MsGRAS19	MsG0480021018.01.T01	5.62	76,258.96	673	Nucleus	Yes	Yes
MsGRAS20	MsG0480021019.01.T01	5.24	83,755.65	735	Nucleus	Yes	Yes
MsGRAS21	MsG0480021020.01.T01	5.5	68,897.38	601	Nucleus	Yes	Yes
MsGRAS22	MsG0480021642.01.T01	5.23	61,704.47	542	Nucleus	Yes	Yes
MsGRAS23	MsG0480021764.01.T01	5.3	50,123.6	452	Nucleus	Yes	Yes
MsGRAS24	MsG0480021771.01.T01	5.91	11,053.79	98	Nucleus	No	Yes
MsGRAS25	MsG0480021850.01.T01	5.2	36,549.71	329	Chloroplast	Yes	Yes
MsGRAS26	MsG0480023446.01.T01	5.58	43,284.82	377	Cytoplasmic	No	No
MsGRAS27	MsG0580024328.01.T01	5.52	54,028.86	481	Cytoplasmic	Yes	Yes
MsGRAS28	MsG0580024757.01.T01	6.1	47,251.93	417	Cytoplasmic	No	No
MsGRAS29	MsG0580027451.01.T05	6.61	30,294.32	271	Chloroplast	No	No
MsGRAS30	MsG0580027451.01.T04	6.52	34,146.74	304	Chloroplast	No	No
MsGRAS31	MsG0580027451.01.T01	5.03	52,231.82	467	Nuclear	No	No
MsGRAS32	MsG0580027451.01.T03	4.86	55,748.52	498	Cytoplasmic	No	No
MsGRAS33	MsG0580027451.01.T02	4.84	56,619.51	505	Cytoplasmic	No	No
MsGRAS34	MsG0580029906.01.T01	5.13	59,194.08	524	Cytoplasmic	Yes	Yes
MsGRAS35	MsG0580030094.01.T01	6	40,152.43	362	Nucleus	Yes	Yes
MsGRAS36	MsG0680032758.01.T01	6.41	74,615.66	671	Chloroplast	Yes	Yes
MsGRAS37	MsG0680033030.01.T01	6.62	74,073.15	666	Chloroplast	Yes	Yes
MsGRAS38	MsG0780037085.01.T01	5.61	75,040.53	676	Nucleus	No	No
MsGRAS39	MsG0780037984.01.T01	5.6	81,306.47	730	Nucleus	Yes	Yes
MsGRAS40	MsG0780037985.01.T01	5.4	81,430.55	731	Nucleus	Yes	Yes
MsGRAS41	MsG0780038783.01.T01	5.93	69,852.63	623	Nucleus	Yes	Yes
MsGRAS42	MsG0780039578.01.T01	6.19	73,091.07	658	Nucleus	Yes	Yes
MsGRAS43	MsG0780041365.01.T01	5.58	64,634.64	556	Nucleus	No	No
MsGRAS44	MsG0880042740.01.T01	5.72	61,482.27	552	Nucleus	Yes	No
MsGRAS45	MsG0880044171.01.T01	4.77	61,196.24	541	Cytoplasmic	Yes	No
MsGRAS46	MsG0880045957.01.T01	5.66	43,074.96	383	Cytoplasmic	Yes	No
MsGRAS47	MsG0880045975.01.T02	5.42	39,808.08	353	Cytoplasmic	No	No
MsGRAS48	MsG0880045975.01.T01	5.5	37,882	335	Cytoplasmic	No	No
MsGRAS49	MsG0880045989.01.T01	4.98	23,447.59	204	Nuclear	No	No
MsGRAS50	MsG0880046208.01.T01	5.51	75,345.09	678	Chloroplast	Yes	No
MsGRAS51	MsG0880047717.01.T01	5.81	60,581.39	540	Nucleus	Yes	Yes

## Data Availability

RNA-seq raw data used in this study was downloaded from the SRA database in NCBI with the Bioproject accession numbers of PRJNA657410 and PRJNA525327.

## Supplementary Materials

The following are available online at https://www.mdpi.com/article/10.3390/ijms22147729/s1, Figure S1: LHRI domain of 51 MsGRAS proteins based on sequence alignment. The most conserved GRAS domain of LHRI was boxed in red; Figure S2: SAW domain of 51 MsGRAS proteins based on sequence alignment. The most conserved GRAS domain of SAW was boxed in red; Figure S3: PFYRE domain of 51 MsGRAS proteins based on sequence alignment. The most conserved GRAS domain of PFYRE was boxed in red; Table S1: Nucleotide and amino acid sequences of MsGRAS; Table S2: The collinearity relationships of the *MsGRAS* genes in four species; Table S3: Analysis and distribution of conserved motifs in MsGRAS proteins; Table S4: The relative expression level of 51 *MsGRAS* genes under various treatments of abiotic stress and treatment time; Table S5: Gene expression level of 51 *MsGRAS* genes in root and leaf tissues under treatments of salt stress; Table S6: The six gene-specific primer pairs used for qRT-PCR validation; Table S7: Expression levels of six *MsGRAS* genes under drought, salt, exogenous ABA, GA and IAA treatments by qRT-PCR; Table S8: Expression data of 51 *MsGRAS* genes in public RNA-seq datasets under stress treatments.

## References

[1] Riano-Pachon D.M., Ruzicic S., Dreyer I., Mueller-Roeber B. (2007). PlnTFDB: An integrative plant transcription factor database. BMC Bioinform..

[2] Hirsch S., Oldroyd G.E. (2009). GRAS-domain transcription factors that regulate plant development. Plant Signal. Behav..

[3] Di Laurenzio L., Wysocka-Diller J., Malamy J.E., Pysh L., Helariutta Y., Freshour G., Hahn M.G., Feldman K.A. (1996). The *SCARECROW* gene regulates an asymmetric cell division that is essential for generating the radial organization of the *Arabidopsis* root. Cell.

[4] Silverstone A.L., Ciampaglio C.N., Sun T.P. (1998). The Arabidopsis RGA gene encodes a transcriptional regulator repressing the gibberellin signal transduction pathway. Plant Cell.

[5] Pysh L.D., Wysocka-Diller J.W., Camilleri C., Bouchez D., Benfey P.N. (1999). The *GRAS* gene family in *Arabidopsis*: Sequence characterization and basic expression analysis of the *SCARECROW-LIKE* genes. Plant J..

[6] Bolle C. (2004). The role of GRAS proteins in plant signal transduction and development. Planta.

[7] Sun X., Jones W.T., Rikkerink E.H. (2012). GRAS proteins: The versatile roles of intrinsically disordered proteins in plant signalling. Biochem. J..

[8] Lee M.-H., Kim B., Song S.-K., Heo J.-O., Yu N.-I., Lee S.A., Kim M., Kim D.G., Sohn S.O., Lim C.E. (2008). Large-scale analysis of the *GRAS* gene family in *Arabidopsis thaliana*. Plant Mol. Biol..

[9] Tian C.G., Wan P., Sun S.H., Li J.Y., Chen M.S. (2004). Genome-wide analysis of the *GRAS* gene family in rice and *Arabidopsis*. Plant Mol. Biol..

[10] Wang L., Ding X., Gao Y., Yang S. (2020). Genome-wide identification and characterization of *GRAS* genes in soybean (*Glycine max*). BMC Plant Biol..

[11] Huang W., Xian Z., Kang X., Tang N., Li Z. (2015). Genome-wide identification, phylogeny and expression analysis of *GRAS* gene family in tomato. BMC Plant Biol..

[12] Liu B., Sun Y., Xue J., Jia X., Li R. (2018). Genome-wide characterization and expression analysis of *GRAS* gene family in pepper (*Capsicum annuum* L.). PeerJ.

[13] Zhang H., Cao Y., Shang C., Li J., Wang J., Wu Z., Ma L., Qi T., Fu C., Bai Z. (2017). Genome-wide characterization of *GRAS* family genes in *Medicago truncatula* reveals their evolutionary dynamics and functional diversification. PLoS ONE.

[14] Wang Y.X., Liu Z.W., Wu Z.J., Li H., Wang W.L., Cui X., Zhuang J. (2018). Genome-wide identification and expression analysis of *GRAS* family transcription factors in tea plant (*Camellia sinensis*). Sci. Rep..

[15] Liu M., Huang L., Ma Z., Sun W., Wu Q., Tang Z., Bu T., Li C., Chen H. (2019). Genome-wide identification, expression analysis and functional study of the *GRAS* gene family in Tartary buckwheat (*Fagopyrum tataricum*). BMC Plant Biol..

[16] Bolle C., Koncz C., Chua N.H. (2000). PAT1, a new member of the *GRAS* family, is involved in phytochrome a signal transduction. Genes Dev..

[17] Song X.M., Liu T.K., Duan W.K., Ma Q.H., Ren J., Wang Z., Li Y., Hou X.L. (2014). Genome-wide analysis of the *GRAS* gene family in Chinese cabbage (*Brassica rapa* ssp. pekinensis). Genomics.

[18] Zhang B., Liu J., Yang Z.E., Chen E.Y., Zhang C.J., Zhang X.Y., Li F.G. (2018). Genome-wide analysis of *GRAS* transcription factor gene family in *Gossypium hirsutum* L.. BMC Genom..

[19] Sidhu N.S., Pruthi G., Singh S., Bishnoi R., Singla D. (2020). Genome-wide identification and analysis of *GRAS* transcription factors in the bottle gourd genome. Sci. Rep..

[20] Yoshida H., Ueguchi-Tanaka M. (2014). DELLA and SCL3 balance gibberellin feedback regulation by utilizing indeterminate domain proteins as transcriptional scaffolds. Plant Signal. Behav..

[21] Lee S.C., Cheng H., King K.E., Wang W.F., He Y.W., Hussain A., Lo J., Harberd N.P., Peng J.R. (2002). Gibberellin regulates *Arabidopsis* seed germination via *RGL2*, a *GAI*/*RGA*-like gene whose expression is up-regulated following imbibition. Genes Dev..

[22] Wen C.K., Chang C. (2002). Arabidopsis *RGL1* encodes a negative regulator of gibberellin responses. Plant Cell.

[23] Peng J., Carol P., Richards D.E., King K.E., Cowling R.J., Murphy G.P., Harberd N.P. (1997). The *Arabidopsis GAI* gene defines a signaling pathway that negatively regulates gibberellin responses. Genes Dev..

[24] Greb T., Clarenz O., Schafer E., Muller D., Herrero R., Schmitz G., Theres K. (2003). Molecular analysis of the lateral suppressor gene in *Arabidopsis* reveals a conserved control mechanism for axillary meristem formation. Genes Dev..

[25] Li M., Wang X., Li C., Li H., Zhang J., Ye Z. (2018). Silencing *GRAS2* reduces fruit weight in tomato. J. Integr. Plant Biol..

[26] Stuurman J., Jaggi F., Kuhlemeier C. (2002). Shoot meristem maintenance is controlled by a *GRAS*-gene mediated signal from differentiating cells. Genes Dev..

[27] Zhang L., Zhu M., Ren L., Li A., Chen G., Hu Z. (2018). The *SlFSR* gene controls fruit shelf-life in tomato. J. Exp. Bot..

[28] Huang W., Peng S., Xian Z., Lin D., Hu G., Yang L., Ren M., Li Z. (2017). Overexpression of a tomato miR171 target gene *SlGRAS24* impacts multiple agronomical traits via regulating gibberellin and auxin homeostasis. Plant Biotechnol. J..

[29] Ma H.S., Liang D., Shuai P., Xia X.L., Yin W.L. (2010). The salt- and drought-inducible poplar GRAS protein SCL7 confers salt and drought tolerance in *Arabidopsis thaliana*. J. Exp. Bot..

[30] Li P., Zhang B., Su T., Li P., Xin X., Wang W., Zhao X., Yu Y., Zhang D., Yu S. (2018). BrLAS, a GRAS transcription factor from *Brassica rapa*, is involved in drought stress tolerance in transgenic *Arabidopsis*. Front. Plant Sci..

[31] Chen H., Zeng Y., Yang Y., Huang L., Tang B., Zhang H., Hao F., Liu W., Li Y., Liu Y. (2020). Allele-aware chromosome-level genome assembly and efficient transgene-free genome editing for the autotetraploid cultivated alfalfa. Nat. Commun..

[32] Li A., Liu A., Du X., Chen J.-Y., Yin M., Hu H.-Y., Shrestha N., Wu S.-D., Wang H.-Q., Dou Q.-W. (2020). A chromosome-scale genome assembly of a diploid alfalfa, the progenitor of autotetraploid alfalfa. Hortic. Res..

[33] Shen C., Du H., Chen Z., Lu H., Zhu F., Chen H., Meng X., Liu Q., Liu P., Zheng L. (2020). The chromosome-level genome sequence of the autotetraploid alfalfa and resequencing of core germplasms provide genomic resources for alfalfa research. Mol. Plant.

[34] Sudhir K., Glen S., Li M., Christina K., Koichiro T. (2018). MEGA X: Molecular evolutionary genetics analysis across computing platforms. Mol. Biol. Evol..

[35] Gruber M.Y., Xia J., Yu M., Steppuhn H., Wall K., Messer D., Sharpe A.G., Acharya S.N., Wishart D.S., Johnson D. (2017). Transcript analysis in two alfalfa salt tolerance selected breeding populations relative to a non-tolerant population. Genome.

[36] Lu X., Liu W., Xiang C., Li X., Wang Q., Wang T., Liu Z., Zhang J., Gao L., Zhang W. (2020). Genome-wide characterization of *GRAS* family and their potential roles in cold tolerance of cucumber (*Cucumis sativus* L.). Int. J. Mol. Sci..

[37] Wang Z., Wang X., Zhang H., Ma L., Zhao H., Jones C.S., Chen J., Liu G. (2020). A genome-wide association study approach to the identification of candidate genes underlying agronomic traits in alfalfa (*Medicago sativa* L.). Plant Biotechnol. J..

[38] Li X., Brummer E.C. (2012). Applied genetics and genomics in alfalfa breeding. Agronomy.

[39] Gao T.W., Zhang W.W., Song A.P., An C., Xin J.J., Jiang J.F., Guan Z.Y., Chen F.D., Chen S.M. (2018). Phylogenetic and transcriptional analysis of chrysanthemum GRAS transcription factors. Biol. Plant..

[40] Lu J., Wang T., Xu Z., Sun L., Zhang Q. (2015). Genome-wide analysis of the *GRAS* gene family in *Prunus mume*. Mol. Genet. Genom..

[41] Xu W., Chen Z., Ahmed N., Han B., Cui Q., Liu A. (2016). Genome-wide identification, evolutionary analysis, and stress responses of the *GRAS* gene family in castor beans. Int. J. Mol. Sci..

[42] Liu X., Widmer A. (2014). Genome-wide comparative analysis of the *GRAS* gene family in *Populus*, *Arabidopsis* and Rice. Plant Mol. Biol. Report..

[43] Moore R.C., Purugganan M.D. (2003). The early stages of duplicate gene evolution. Proc. Natl. Acad. Sci. USA.

[44] Tang H., Wang X., Bowers J.E., Ming R., Alam M., Paterson A.H. (2008). Unraveling ancient hexaploidy through multiply-aligned angiosperm gene maps. Genome Res..

[45] Cannon S.B., Mitra A., Baumgarten A., Young N.D., May G. (2004). The roles of segmental and tandem gene duplication in the evolution of large gene families in *Arabidopsis thaliana*. BMC Plant Biol..

[46] Zou M., Guo B., He S. (2011). The roles and evolutionary patterns of intronless genes in deuterostomes. Comp. Funct. Genom..

[47] Jain M., Nijhawan A., Arora R., Agarwal P., Ray S., Sharma P., Kapoor S., Tyagi A.K., Khurana J.P. (2007). F-box proteins in rice. Genome-wide analysis, classification, temporal and spatial gene expression during panicle and seed development, and regulation by light and abiotic stress. Plant Physiol..

[48] Jain M., Tyagi A.K., Khurana J.P. (2006). Genome-wide analysis, evolutionary expansion, and expression of early auxin-responsive *SAUR* gene family in rice (*Oryza sativa*). Genomics.

[49] Aubourg S., Kreis M., Lecharny A. (1999). The DEAD box RNA helicase family in *Arabidopsis thaliana*. Nucleic Acids Res..

[50] Zhang D., Iyer L.M., Aravind L. (2012). Bacterial GRAS domain proteins throw new light on gibberellic acid response mechanisms. Bioinformatics.

[51] Li M.Y., Xu Z.S., Tian C., Huang Y., Wang F., Xiong A.S. (2016). Genomic identification of *WRKY* transcription factors in carrot (*Daucus carota*) and analysis of evolution and homologous groups for plants. Sci. Rep..

[52] Dill A., Jung H.S., Sun T.P. (2001). The DELLA motif is essential for gibberellin-induced degradation of *RGA*. Proc. Natl. Acad. Sci. USA.

[53] King K.E., Moritz T., Harberd N.P. (2001). Gibberellins are not required for normal stem growth in *Arabidopsis thaliana* in the absence of *GAI* and *RGA*. Genetics.

[54] Harberd N.P. (2003). Relieving DELLA restraint. Science.

[55] Achard P., Vriezen W.H., Van Der Straeten D., Harberd N.P. (2003). Ethylene regulates *Arabidopsis* development via the modulation of DELLA protein growth repressor function. Plant Cell.

[56] Aoyanagi T., Ikeya S., Kobayashi A., Kozaki A. (2020). Gene regulation via the combination of transcription factors in the indeterminate domain and *GRAS* families. Genes.

[57] Heo J.O., Chang K.S., Kim I.A., Lee M.H., Lee S.A., Song S.K., Lee M.M., Lim J. (2011). Funneling of gibberellin signaling by the *GRAS* transcription regulator scarecrow-like 3 in the *Arabidopsis* root. Proc. Natl. Acad. Sci. USA.

[58] Liang W.H., Shang F., Lin Q.T., Lou C., Zhang J. (2014). Tillering and panicle branching genes in rice. Gene.

[59] Schumacher K., Schmitt T., Rossberg M., Schmitz G., Theres K. (1999). The Lateral suppressor (Ls) gene of tomato encodes a new member of the VHIID protein family. Proc. Natl. Acad. Sci. USA.

[60] Agarwal P., Jha B. (2010). Transcription factors in plants and ABA dependent and independent abiotic stress signalling. Biol. Plant..

[61] Suzuki N., Koussevitzky S., Mittler R., Miller G. (2012). ROS and redox signalling in the response of plants to abiotic stress. Plant Cell Environ..

[62] Navarro L., Bari R., Achard P., Lison P., Nemri A., Harberd N.P., Jones J.D. (2008). DELLAs control plant immune responses by modulating the balance of jasmonic acid and salicylic acid signaling. Curr. Biol..

[63] Xu K., Chen S., Li T., Ma X., Liang X., Ding X., Liu H., Luo L. (2015). *OsGRAS23*, a rice GRAS transcription factor gene, is involved in drought stress response through regulating expression of stress-responsive genes. BMC Plant Biol..

[64] Finn R.D., Bateman A., Clements J., Coggill P., Eberhardt R.Y., Eddy S.R., Heger A., Hetherington K., Holm L., Mistry J. (2014). Pfam: The protein families database. Nucleic Acids Res..

[65] Chen C., Chen H., Zhang Y., Thomas H.R., Frank M.H., He Y., Xia R. (2020). TBtools: An integrative toolkit developed for interactive analyses of big biological data. Mol. Plant.

[66] Wang Y., Tang H., Debarry J.D., Tan X., Li J., Wang X., Lee T.H., Jin H., Marler B., Guo H. (2012). MCScanX: A toolkit for detection and evolutionary analysis of gene synteny and collinearity. Nucleic Acids Res..

[67] Bailey T.L., Boden M., Buske F.A., Frith M., Grant C.E., Clementi L., Ren J., Li W.W., Noble W.S. (2009). MEME SUITE: Tools for motif discovery and searching. Nucleic Acids Res..

[68] Subramanian B., Gao S., Lercher M.J., Hu S., Chen W.H. (2019). Evolview v3: A webserver for visualization, annotation, and management of phylogenetic trees. Nucleic Acids Res..

