# Supplementary figures and images for: Genome-Wide Identification of GRAS Gene Family and Their Responses to Abiotic Stress in Medicago sativa
Source: Int J Mol Sci. 2021 Jul 20;22(14):7729. doi: 10.3390/ijms22147729 (PMC8304046; doi:10.3390/ijms22147729)

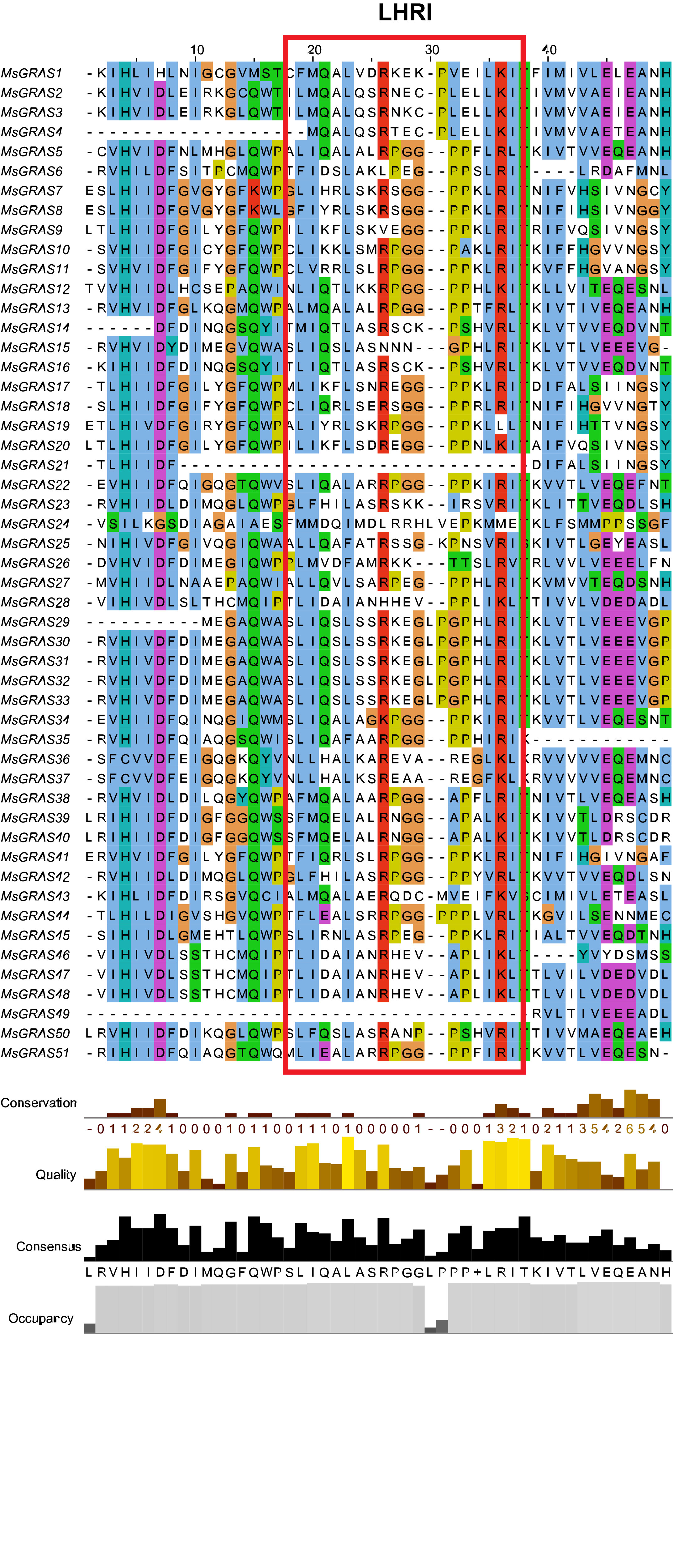

Supplement: Supplementary file 1 [file ijms-22-07729-s001.zip › supps resubmitted 2/Fig.S1.jpg]

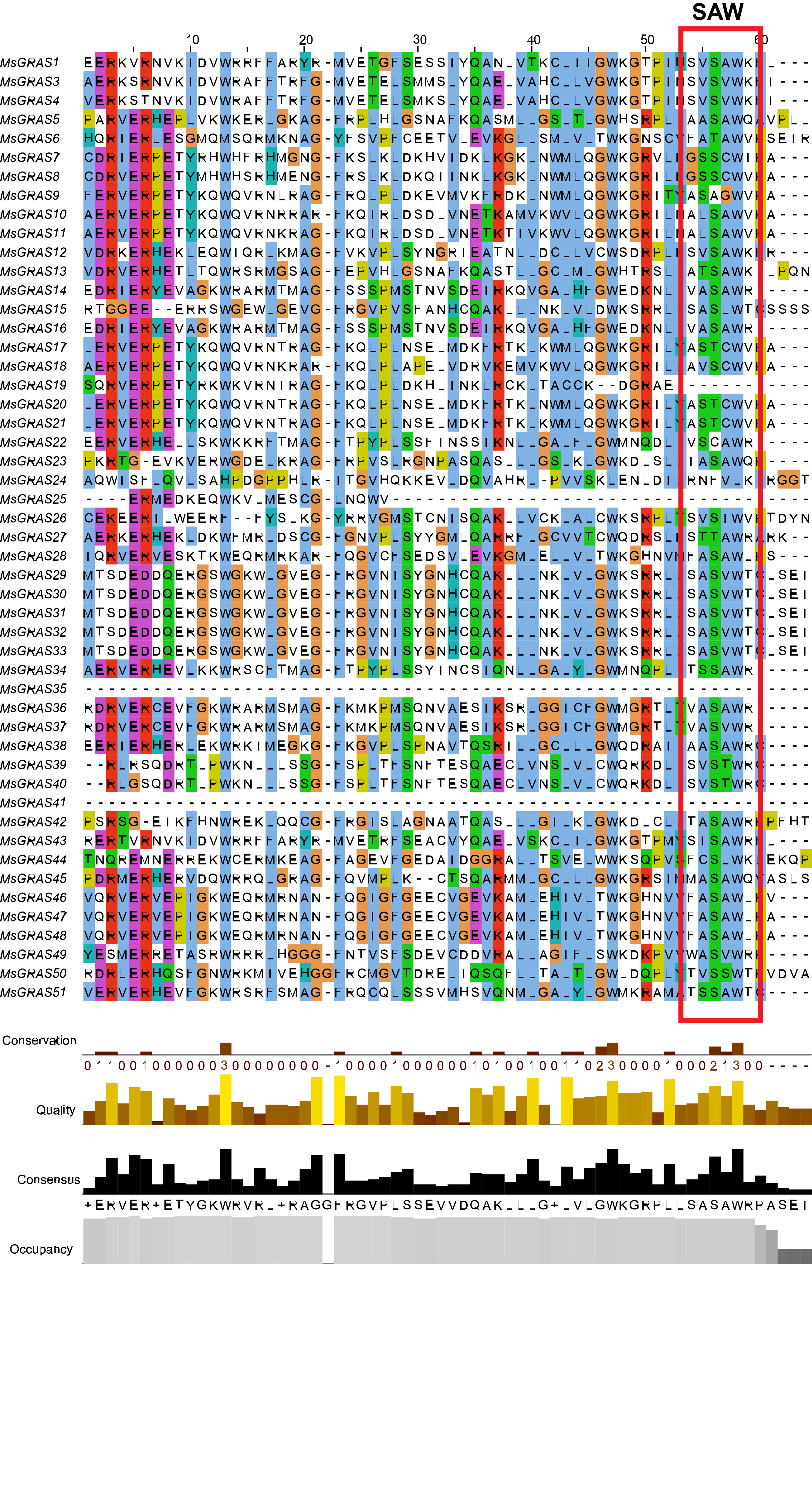

Supplement: Supplementary file 1 [file ijms-22-07729-s001.zip › supps resubmitted 2/Figure S2.jpg]

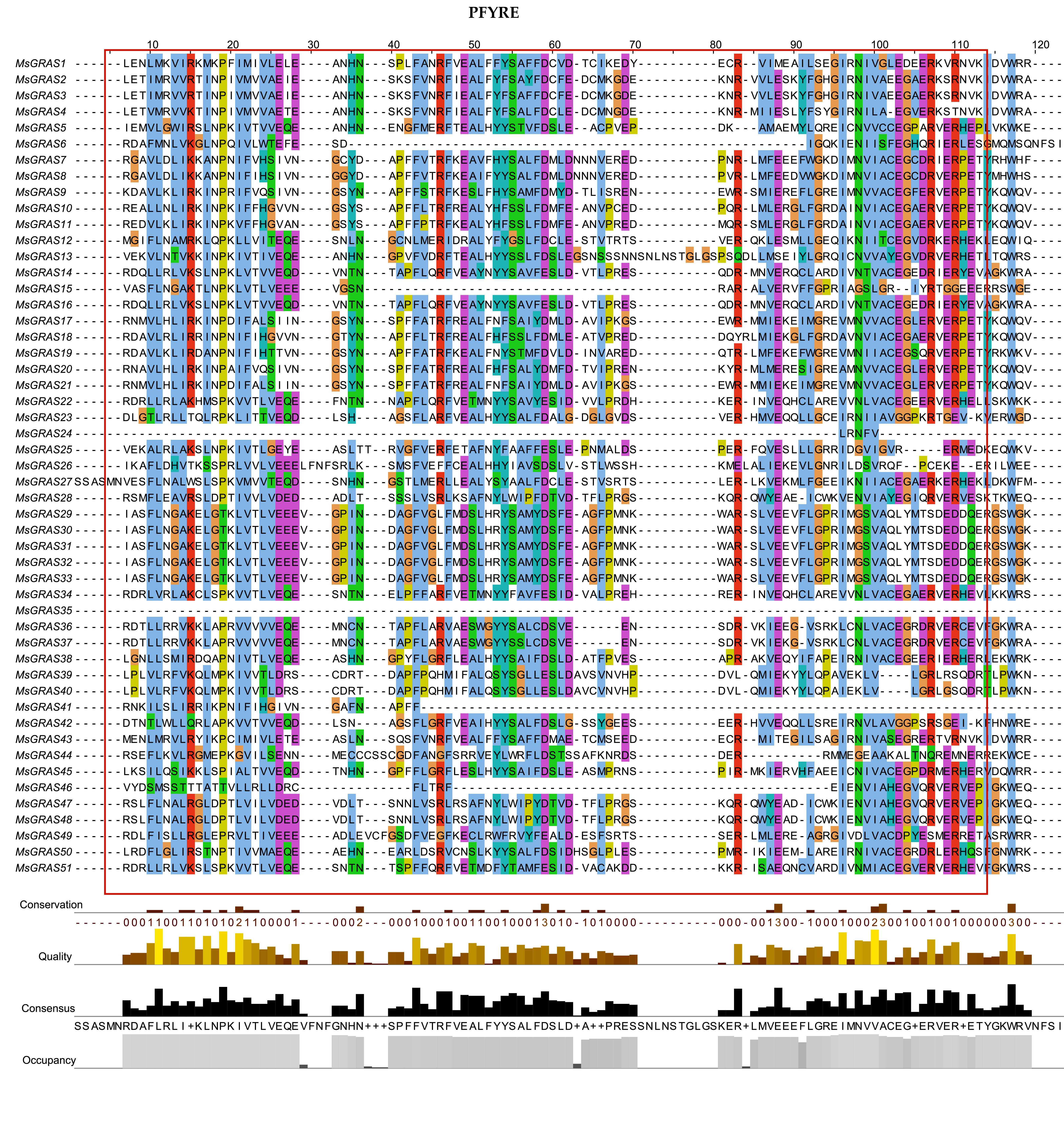

Supplement: Supplementary file 1 [file ijms-22-07729-s001.zip › supps resubmitted 2/Figure S3.jpg]
